# Oxidized LDL Is Associated with eGFR Decline in Proteinuric Diabetic Kidney Disease: A Cohort Study

**DOI:** 10.1155/2021/2968869

**Published:** 2021-10-19

**Authors:** Stefanos Roumeliotis, Athanasios Roumeliotis, Panagiotis I. Georgianos, Aikaterini Stamou, Vangelis G. Manolopoulos, Stylianos Panagoutsos, Vassilios Liakopoulos

**Affiliations:** ^1^Division of Nephrology and Hypertension, 1^st^ Department of Internal Medicine, AHEPA Hospital, School of Medicine, Aristotle University of Thessaloniki, 54636 Thessaloniki, Greece; ^2^Department of Microbiology, AHEPA Hospital, School of Medicine, Aristotle University of Thessaloniki, 54636 Thessaloniki, Greece; ^3^Laboratory of Pharmacology, Medical School, Democritus University of Thrace, 68100 Alexandroupolis, Greece; ^4^Department of Nephrology, Medical School, Democritus University of Thrace, 68100 Alexandroupolis, Greece

## Abstract

Diabetic kidney disease (DKD) is a highly heterogenous disease, including the proteinuric and the nonproteinuric pattern. Oxidized low-density lipoprotein (ox-LDL) is progressively increased in DKD and causes direct damage to kidney tubular epithelial cells through a mechanism similar to that underlying the deleterious effect of lipid peroxides in the vascular endothelium. We aimed to examine the association between plasma ox-LDL cholesterol and clinical endpoints in DKD patients. Ninety-one patients with established proteinuric DKD and diabetic retinopathy were enrolled and prospectively followed for 10 years or the occurrence of death, or at least 30% decline in eGFR, or progression to end-stage kidney disease (ESKD) requiring renal replacement therapy (primary outcome). At the end of the study, both eGFR and proteinuria were reassessed. Secondary outcomes of the study were the percentage change in eGFR and proteinuria over time for each patient. At baseline, patients were divided into 2 groups according to the median ox-LDL value (i.e., below or equal and above 66.22 U/L). Both Kaplan-Meier curves (*p* = 0.001, log-rank test) and univariate Cox regression analysis showed that high ox-LDL was associated with the primary outcome (HR = 3.42, 95%CI = 1.55 − 7.56, *p* = 0.002). After adjustment for various well-known cofounders, multivariate Cox analysis showed that the association between increased circulating ox-LDL levels and the composite kidney endpoint remained significant (HR = 2.87, 95%CI = 1.14–7.20, *p* = 0.025). Regarding the secondary outcome of eGFR decline, the assessment of areas under the curves (AUC) showed that ox-LDL outperformed several cofounding factors (AUC 71%, 95%CI = 0.59 − 0.83, *p* = 0.001) and had better accuracy to predict deterioration of eGFR over time than baseline proteinuria (AUC 67%, 95%CI = 0.54 − 0.79, *p* = 0.014). Increased ox-LDL might be associated with disease progression in proteinuric DKD.

## 1. Introduction

Diabetic kidney disease (DKD) presents in approximately 40% of type 2 diabetes mellitus (T2DM) subjects over lifetime and remains the leading cause of end-stage kidney disease (ESKD) worldwide [1]. Since DKD is an heterogenous disease, including both proteinuric and nonproteinuric progressive patterns, scientific research has focused on the identification of factors promoting progression of the disease [2]. Impaired lipid profile, including increased triglycerides, low-density lipoprotein (LDL), and/or decreased high-density lipoprotein (HDL) particles, is a common complication of progressive DKD and contributes to the heavy cardiovascular (CV) burden that characterizes DKD patients. Dyslipidemia might cause severe injury in podocytes, mesangial, and glomerular endothelial cells, whereas lipid-lowering therapy has been shown to ameliorate kidney damage [3].

Due to the imbalance between pro and antioxidants in favor of the former, oxidative stress (OS) is present even at early stages of DKD and progresses along with disease deterioration to ESKD [4, 5]. OS promotes the oxidative conversion of LDL to oxidized LDL (ox-LDL) within the arterial wall, which is the first, key event towards development of atherosclerosis. Although ox-LDL is a marker of OS, endothelial dysfunction (ED) and atherosclerosis and these entities have been repeatedly reported to be progressively accelerated with deterioration of CKD, the clinical studies assessing the association between ox-LDL and clinical hard endpoints in DKD remain limited and thus up to date, ox-LDL have not been established as a biomarker in DKD. Moreover, strategies to ameliorate OS and dyslipidemia in both T2DM and CKD populations have failed to show any improvement on clinical hard endpoints. Although experimental data suggest that ox-LDL might play a pivotal role in the pathogenesis of tubulointerstitial lesions, glomerulopathy, and podocytopathy in proteinuric DKD [6, 7], until to date, no study has assessed the possible association between circulating ox-LDL and kidney outcomes in proteinuric DKD patients.

Aim of this study was to examine the association between plasma ox-LDL cholesterol and mortality, deterioration of kidney function, and proteinuria increase in a cohort of patients with established proteinuric DKD.

## 2. Materials and Methods

### 2.1. Patients

A total of 91 patients with established DKD were enrolled from the Diabetic Chronic Kidney Disease Department's outpatient clinic of the General University of Alexandroupolis in Greece. Participants provided their written, informed consent at recruitment. The study protocol was approved by the PhD Scientific Committee (1046/01 November 2007) and further approved by the Ethics Committee of the Scientific Council of the University General Hospital of Alexandroupolis (1130/25 November 2011) and was in accordance with the Helsinki Declaration of Human Rights. Eligible patients for enrolment were those who had a history of T2DM of at least 7 years, presence of diabetic retinopathy, and CKD, defined by persistent proteinuria and/or eGFR below 90 ml/min. The exclusion criteria included acute illness, chronic inflammatory disease, urinary tract disease, laboratory or clinical evidence of nondiabetic CKD, and documented transitory of permanent eGFR decline > 30% during the last 6 months before recruitment. At baseline, we documented somatometric, biochemical and clinical data and history of smoking habit, T2DM duration, and previous CV disease. Definition and classification of DKD stages were done according to the clinical practice guidelines [8] established by the Kidney Disease Improving Global Outcomes (KDIGO) in 2012 and eGFR, was calculated by the CKD epidemiology collaboration (CKD-EPI) equation.

### 2.2. Follow-Up and Endpoints

After enrolment, all participants were prospectively followed for a period of 10 years (from 20 November 2008 to 21 November 2018), or the occurrence of death, or at least 30% decline in eGFR, or progression to ESKD requiring renal replacement therapy (primary outcome). At the end of the study, both eGFR and UPCR were reassessed. Secondary outcomes of the study were the percentage change in eGFR and UPCR over time (calculated as *Δ*eGFR/baseline eGFR and *Δ*UPCR/baseline UPCR, respectively) divided by the follow-up time) for each patient. Follow-up data were collected from regular follow-up visits, death certificates, and via integrated telephone interviews.

### 2.3. Laboratory Analyses

The laboratory methods for this study have been extensively described elsewhere [9]. We collected fasting blood samples from all participants to obtain whole blood, serum and plasma. Serum and whole blood samples were transferred to the laboratory for analysis, whereas plasma samples were immediately centrifuged and stored at −20°C until analysis. Plasma ox-LDL concentrations were determined by a capture ELISA using sandwich antibodies (human ox-LDL ELISA kit, Mercodia, Sweden). According to the manufacturers, detection limit for ox-LDL assays was 0.3 U/L, and intra/interassay coefficients of variation were <10%. Proteinuria was evaluated as the urine protein to creatinine ratio (UPCR) in 3 consecutive morning spot urine samples, using the turbidimetric immunoassay, as described before [10].

### 2.4. Statistical Analysis

To evaluate the association between plasma ox-LDL and our study outcomes, at baseline, patients were divided into 2 groups according to the median ox-LDL value (i.e., below or equal and above 66.22 U/L). Data were tested for normality with the Kolmogorov–Smirnov test, and normally distributed continuous variables were expressed as the mean (standard deviation), whereas nonnormally continuous variables were expressed as (range) and binary variables as percent frequency. Differences of variables among groups were explored using the chi-square test for frequencies (dichotomized variables) and the Mann–Whitney test for continuous variables. We used the Kaplan–Meier actuarial method and the log-rank test to compare survival curves to estimate the primary outcome (all-cause mortality or eGFR decline of at least 30% from baseline or progression to ESKD requiring dialysis) for the median plasma ox-LDL level. Univariate and multivariate Cox proportional hazard analysis (forward stepwise regression) was performed to evaluate adjusted hazard ratios (HRs) and 95% confidence intervals (Cis) for the associations between groups of ox-LDL and the primary, composite outcome. Multivariate models were adjusted for all variables that were associated with the primary outcome in univariate models (serum albumin, triglycerides, baseline UPCR, and baseline eGFR). The multivariate Cox proportional hazard analysis for the primary, composite outcome was also performed in 500 bootstrap samples.

To further examine the possible effect of ox-LDL on our secondary endpoint (the percentage change in eGFR and proteinuria over time, calculated as *Δ*eGFR/baseline eGFR and *Δ*proteinuria/baseline proteinuria, respectively, and divided by the follow-up time for each patient), after the end of the follow-up period, we categorized our cohort to slow and fast decliners. Mann–Whitney *U* test and chi-square test were used to identify differences of all the variables assessed at baseline (in Table 1) among fast and slow decliners. We then performed a statistical analysis with receiver operation curves (ROC) to examine the association of the variables that were significantly different among groups of decliners (baseline UPCR, ox-LDL, triglycerides, duration of T2DM, and background history of CV disease). In conjunction with ROC curves, to determine the optimal cut off value of ox-LDL to predict the percentage eGFR change over time, we calculated the Youden index. Statistical analyses were performed using the IBM Statistical Package for Social Sciences (SPSS 18.0 for Windows, Chicago, IL, USA). Significance was set at *p* < 0.05.

## 3. Results

### 3.1. Baseline Characteristics

The study population included 91 DKD subjects (mean age: 67.16 ± 8.2 years, females: 52.7%), with T2DM for a median time of 13 years and median eGFR 59.6 ml/min and UPCR 0.15 g/g at baseline, at different stages of CKD: 19 at CKD stage 1, 26 at CKD stage 2, 23 at CKD stage 3, and 12 at CKD stage 4. At baseline, 41/91 patients had normal to mildly increased albuminuria (A1 stage), 33 moderately increased (A2 stage), and 17 severely increased albuminuria (A3 stage). Among CKD stages, A1 was more prevalent at CKD stage 1 (89.5%) and A3 at CKD stage 4 (75%). Participants were mainly overweight and obese, with a mean body mass index (BMI) of 31.4 ± 5.4 kg/m^2^, and had acceptable blood pressure and glycemic control, as assessed by median systolic/diastolic blood pressure (SBP/DBP) of 140/80 mm Hg and median glycated hemoglobin (HbA1c) of 7.2%, whereas the majority of patients (70.3%) had history of CV disease. The median value of ox-LDL was 66.22 (22.9-123.4 U/L) at baseline. The baseline demographic, somatometric, and clinical characteristics of the study cohort according to median plasma ox-LDL levels are shown in Table 1. Although the two groups did not differ significantly in gender, age, smoking habit, history of CV disease, duration of T2DM, BMI, SBP, hemoglobin, serum albumin, HBA1c, c-reactive protein (CRP), HDL cholesterol, and UPCR, patients in the high ox-LDL group presented higher DBP and lower eGFR values, at baseline. As expected, compared to the low, patients in the high ox-LDL group had higher total/LDL cholesterol and triglycerides levels.

### 3.2. Outcomes

**Figure 1:**
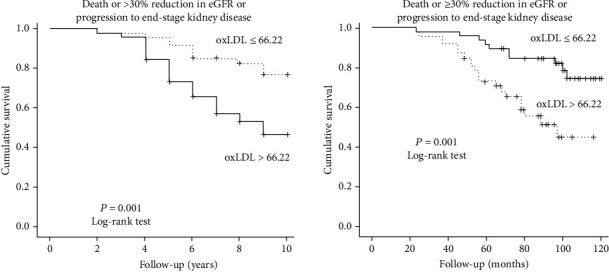
Kaplan-Meier curves for mortality or eGFR decline ≥ 30% or progression to ESRD in DKD patients with high and low plasma ox-LDL levels [according to the median value (66.22 U/L)]. Log − rank test *p* = 0.001.

**Figure 2:**
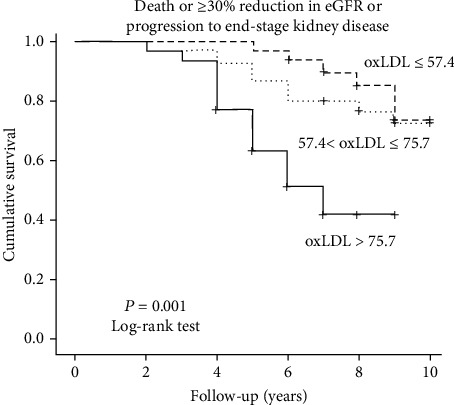
Kaplan-Meier curves for mortality or eGFR decline ≥ 30% or progression to ESRD in DKD patients with high, moderate, and low plasma ox-LDL levels (3 groups according to ox-LDL levels (≤57.4 U/L, >57.4, ≤75.7 U/L, and >75.7 U/L)). Log − rank test *p* < 0.001.

During the follow-up period (median: 8 years; range: 2 to 10 years), 30/91 patients presented the composite endpoint (13 died and 17 presented deterioration of eGFR ≥ 30% or progression to ESKD requiring dialysis), 10 in the low, and 20 in the high ox-LDL group. The prevalence of our primary outcome increased progressively among CKD stages (10.5%, 26.9%, 47.8%, and 83.3% in CKD stages 1, 2, 3 and 4, respectively). Fast eGFR decliners were the 21% of patients with CKD stage 1 and 36.4%, 65.5%, and 100% of patients with stages 2, 3, and 4, respectively. Kaplan-Meier curves showed that patients with circulating Ox-LDL cholesterol above the median (>66.22 U/L) had a significantly higher risk for the composite outcome (*p* = 0.001, log-rank test, Figure 1). At baseline, eGFR was significantly different among high and low ox-LDL groups. To overcome this limitation, we further divided our cohort to 3 groups according to ox-LDL levels (≤57.4 U/L, >57.4 and ≤75.7 U/L and >75.7 U/L). Kruskal-Wallis test showed that eGFR did not significantly differ among these 3 groups (*p* = 0.17). We performed Kaplan-Meier analysis again and found that patients with high ox-LDL levels (3rd group) had significantly increased risk for the primary outcome (*p* < 0.0001, log-rank test, Figure 2). Univariate Cox proportional hazard analysis showed that high plasma ox-LDL levels were independently associated with the composite endpoint of mortality or 30% eGFR reduction or progression to ESKD ((HR) = 3.42, 95%CI = 1.55 − 7.56, *p* = 0.002), Table 2. In univariate models, baseline eGFR (HR = 0.97, 95%CI = 0.95 − 0.98, *p* < 0.001), baseline UPCR (HR = 1.43, 95%CI = 1.12 − 1.81, *p* = 0.004), triglycerides (HR = 1.01, 95%CI = 1.00 − 1.01, *p* = 0.007), and serum albumin (HR = 0.35, 95%CI = 0.16 − 0.79, *p* = 0.011) were the only factors associated with the study outcome, whereas the association with duration of T2DM was marginally nonsignificant (*p* = 0.057). After adjustment for these well-known cofounders, multivariate Cox analysis showed that the association between increased circulating ox-LDL levels and the study endpoint remained significant (HR = 2.87, 95%CI = 1.14–7.20, *p* = 0.025), whereas the only other factor associated with the study outcome was baseline eGFR (HR = 0.98, 95%CI = 0.96–0.99, *p* = 0.013), Table 2. To verify the results in a larger population, we performed the original multivariate Cox analysis in 500 bootstrap samples and found that the association between high ox-LDL and the composite outcome remained significant (HR = 2.70, 95%CI = 1.02 − 7.16, *p* = 0.046, Supplementary Table S1), whereas the association with baseline eGFR was lost.

**Figure 3:**
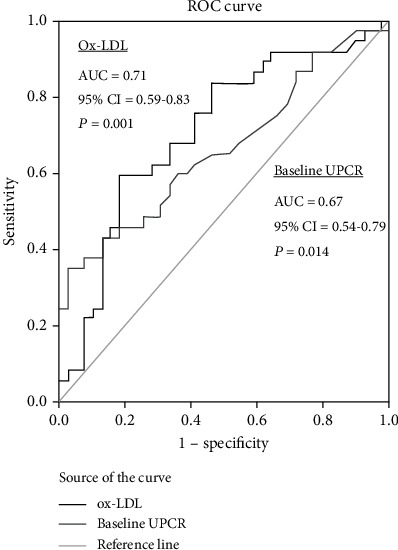
Receiver operating characteristic curves showing the performance of ox-LDL and baseline UPCR in predicting eGFR decline over time, in DKD patients.

After the follow-up period of 10 years, 22 patients had stable or increased kidney function, whereas 56 experienced reduction of eGFR. Patients were divided in two groups according to the calculated eGFR decrease over the time period: fast decliners (those who had above the median eGFR change over time) and slow decliners.  Table 3 shows that compared to slow, fast decliners had significantly increased prevalence of CV disease history (*p* = 0.023, chi-square test), increased duration of T2DM, higher triglycerides and ox-LDL levels, and higher baseline UPCR values (*p* = 0.028, *p* = 0.004, *p* = 0.005, *p* = 0.013, respectively, Mann–Whitney test). After testing the predictive performance of all these factors associated with fast eGFR decline in Table 3, the assessment of areas under the curves (AUC) showed that Ox-LDL outperformed the other factors (AUC 71%, 95%CI = 0.59 − 0.83, *p* = 0.001, Figure 3) and had better accuracy to predict deterioration of eGFR over time to baseline UPCR (AUC 67%, 95%CI = 0.54 − 0.79, *p* = 0.014, Figure 3). Youden index revealed that the optimal cut-off value of ox-LDL for predicting the percentage eGFR change over time was 73.1 U/L. No association was found between ox-LDL and decline of UPCR over time.

## 4. Discussion

In this study, we aimed to assess whether ox-LDL, a biomarker of OS and ED, is associated with progression of DKD, in a cohort of 91 patients with established proteinuric DKD. Our outcomes of interest (all-cause mortality, incident ESKD, ≥30% reduction of baseline eGFR and decline rate in kidney function and proteinuria) have been widely used as validated endpoints in several prospective studies before [11–14]. The annual eGFR decline varied significantly among these studies. This could be partially attributed to different study design, cohort, study duration, and selection criteria; however, the high variability in eGFR decline highlights the necessity of determining novel biomarkers that could predict progression of DKD.

In our study, after a 10-year follow-up, 22 patients had stable kidney function, whereas 56 experienced reduction of eGFR, similarly to previous reports [13, 15]. *Ι*t is hypothesized that the main pathogenetic mechanism driving progression of nonproteinuric DKD is macroangiopathy, whereas microangiopathy is responsible for progression of proteinuric DKD. We found that proteinuria was associated with the primary composite outcome of mortality, at least 30% reduction in eGFR or EKSD in univariate analysis, but this association was lost in multivariate analysis. Moreover, proteinuria predicted eGFR decline. Our findings are in line with data from large epidemiological studies showing that eGFR and proteinuria are independent predictors of kidney outcomes in T2DM patients [14, 16]. Furthermore, in a prospective longitudinal study of 1,984 T2DM subjects, it was reported that the degree of proteinuria affected the rate of eGFR decline over time, with high proteinuric patients manifesting the steepest eGFR reduction, over the follow-up period of 16 years [17].

The main finding of our study was that among patients with established proteinuric DKD and presence of retinopathy, circulating ox-LDL was associated with the composite renal outcome of all-cause mortality, at least 30% reduction of eGFR and/or progression to ESKD. Moreover, ox-LDL predicted eGFR decline over the follow-up period of 10 years with better accuracy than proteinuria. There is a growing body of evidence suggesting that hyperglycemia and uremia independently trigger accumulation of reactive oxygen species (ROS) and depletion of antioxidant defense mechanisms [4, 5, 18]. As DKD progresses, OS promotes accumulation of free radicals to decrease nitric oxide bioavailability that is necessary for vascular relaxation. As a result, the arterial wall LDL particles undergo oxidative modification to form ox-LDL, which lead to formation of malondialdehyde (MDA), a highly reactive lipid peroxide [19]. In turn, MDA triggers adhesion of inflammatory cytokines and macrophages which bind to ox-LDL particles to form foam cells. Through the mechanism of ox-LDL activation, OS promotes ED, the hallmark of atherosclerosis in T2DM and CKD patients [20]. Several studies have showed that ox-LDL is an independent determinant of ED in both CKD and HD populations [21, 22], whereas the hyperglycemic environment along with ox-LDL triggers overproduction of asymmetric dimethylarginine (ADMA) which in turn promotes endothelial nitric oxide synthase uncoupling to form ROS [20]. In T2DM patients, both ADMA and limited nitric oxide bioavailability, independently from each other, predict micro and macrovascular complications, including onset of DKD [23]. Moreover, the rs11780592 polymorphism of the soluble epoxide hydrolase 2 (EPHX2), an enzyme triggering cellular apoptosis through induction of OS and inflammation, was associated with ox-LDL, albuminuria, and mortality in a cohort of DKD patients [24]. But even locally, experimental data suggest that ox-LDL might play a key role in the development of glomerulosclerosis in DKD. In experimental uremia and hypreglycemia, an excessive uptake of ox-LDL in podocytes, renal tubular, renal epithelial, and mesangial cells is reported, leading to intrarenal accumulation of lipid peroxides and loss of nephrin [25]. These oxidative modifications of all types of renal cells are leading to progressive alterations of their structure and function and therefore result to diabetic glomerulopathy [26]. There is a growing body of in vitro and in vivo data suggesting that experimental uremia triggers accumulation of ox-LDL in renal tissues and diet-derived increase in circulating ox-LDL promotes fibrosis, glomerulosclerosis, and loss of kidney function [27–29]. In agreement with these data, a kidney biopsy study in kidney transplant recipients after 1.5 years of follow-up showed that interstitial accumulation of ox-LDL was associated with the presence of tubulointerstitial macrophages and the development of interstitial fibrosis and subsequent glomerulosclerosis [30]. In another cross-sectional study, Solatani et al. [31] enrolled 58 ESRD patients undergoing HD that would receive a kidney transplant and found that ox-LDL levels were inversely correlated with the restored kidney function after transplantation and positively with the level of immunosuppressive therapy (cyclosporine). An experimental study showed that in DKD patients, the upregulated ox-LDL expression in podocytes and primary tubular cells induced fibrosis and might be an early event in the onset of the disease [26]. Data from epidemiological studies strongly support that dyslipidemia is an independent factor associated with both development and progression of CKD and DKD [32–34]. However, it became evident that ox-LDL and not native LDL is responsible for the kidney injury in tubular cells and podocytes, via a pathogenetic mechanism that is similar to that underlying the toxicity of lipid peroxides to the vascular endothelium [7]. In agreement with this, we found that ox-LDL was associated with kidney clinical outcomes, irrespectively of LDL levels and other lipid parameters.

Several clinical studies are aimed at assessing the possible association between ox-LDL and DKD; however, the majority of these studies had cross-sectional design and focused only on proteinuria. In a cohort of 70 T2DM patients, Ujihara et al. showed that compared to those with normo and microalbuminuria, patients with macroalbuminuria exhibited significantly higher plasma ox-LDL levels, regardless of glycemic control [35]. The authors hypothesized that ox-LDL might play a role in the progression of diabetic nephropathy. Another cohort study in T2DM patients showed that compared to normoalbuminuric, patients with microalbuminuria had increased LDL oxidation, which was attributed ti the suboptimal metabolic control and increased triglycerides in these patients [36]. Data from the Diabetes Control and Complications Trial showed that in a cohort of 518 T1DM patients with various degrees of albuminuria, followed for 14-20 years, increased levels of ox-LDL in circulating immune complexes at baseline were significantly associated with higher odds for development of macroalbuminuria [37]. Every increase of 1 SD in ox-LDL levels was associated with 2.5 times higher odds ratio for macroalbuminuria development, even after adjustment for diabetic retinopathy, diabetes duration, glycated hemoglobin, and LDL. However, the same group of investigators found that ox-LDL in immune complexes from serum failed to associate with kidney outcomes, including development of macroalbuminuria in T2DM patients [38]. In our study, we found that circulating ox-LDL was associated with decline of the kidney function but not proteinuria increase, in a cohort of T2DM patients with proteinuric DKD. In agreement with the study by Lopes-Virella, we also found that the association between ox-LDL and progression of DKD was independent of diabetes duration, HBA1c, and LDL levels.

In two cross-sectional studies, circulating ox-LDL was not significantly increased in ESRD patients (either on HD or peritoneal dialysis) compared to predialysis CKD [39, 40]. However, compared to controls with the normal kidney function, CKD patients exhibit a nearly 10-fold increase in ox-LDL levels, thus indicating a high OS status in these patients [40]. Moreover, we found that the cut-off value of ox-LDL to predict eGFR decline was 73.1 U/L, which is a relatively high value, and thus we hypothesize that increased OS might be associated with deterioration of the kidney function in DKD. In agreement with our study, Morales-Indiano conducted a prospective study and enrolled 131 HD patients (with and without T2DM) who underwent kidney transplantation and assessed markers of inflammation and OS (including anti-ox-LDL antibodies) at baseline and 12 months after transplantation [41]. The authors found a significant negative correlation between anti-ox-LDL antibodies and kidney function after 1 year of kidney transplantation; this association was more pronounced among diabetics. Furthermore, we recently showed that ox-LDL might amplify the tight association between proteinuria and deterioration of kidney function in proteinuric DKD [42].

The majority of the forementioned studies are either experimental or cross-sectional and therefore are limited by design to assess the predictive value of ox-LDL on clinical hard endpoints. The accumulated data suggesting a pivotal role of OS on development of DKD encouraged research on the possible renoprotective effect of antilipidemic and antioxidant supplementation. Although antioxidant treatment in experimental studies generated favorable results [43], clinical data are limited and contraindicatory [19, 44]. Treatment with antilipidemic agents improved ED through a decrease in ox-LDL levels [45] and successfully prevented ox-LDL-mediated injury of glomerular podocytes and thus might exert antiproteinuric and renoprotective effects [46]. In a cohort of 31 CKD patients in stages 3 and 4, correction of metabolic acidosis with sodium bicarbonate supplementation for 1 year successfully prevented progression of CKD and decreased electronegative LDL (a minimally ox-LDL molecule) plasma levels [47]. These data suggest a possible role of ox-LDL in the development of CKD.

To our knowledge, this is the first study showing that circulating ox-LDL might be a predictor of DKD progression in patients with proteinuric DKD. However, our study has certain limitations. The observational design cannot establish any causality, and we did not measure any other markers of lipid peroxidation status and circulating antioxidants. Moreover, the relatively small sample size is also recognized as limitation; although, several studies with similar design and population have used similar sample sizes. The evaluation of ox-LDL as a potential biomarker for the development of DKD still remains for research.

## 5. Conclusions

In conclusion, our study demonstrated that ox-LDL, a marker of OS and ED, might be associated with mortality and deterioration of the kidney function in a cohort of proteinuric DKD patients with diabetic retinopathy. No association was found between ox-LDL and proteinuria increase in these patients. Future, larger randomized controlled studies targeting the ox-LDL suppression are needed in order to draw definite conclusions regarding the possible association between ox-LDL and deterioration of DKD.

## Figures and Tables

**Table 1:** Baseline anthropometric, clinical, and biochemical data of patients with diabetic kidney disease, below and above median plasma oxidized LDL levels. Results for continuous variables are presented as mean (S.D.) or median (range).

	Ox − LDL ≤ 66.22 U/L (*n* = 46)	Ox − LDL > 66.22 U/L (*n* = 45)	All patients (*n* = 91)	*p*
Age (years)	66.3 ± 8.1	68.1 ± 8.3	67.2 ± 8.2	0.33
Gender (M/F)	20/26	23/22	43/48	0.30
Smoking habit (%)	19%	26.6%	23,1%	0.29
History of CV disease (%)	65%	75.5%	70.3%	0.20
Duration of T2DM (years)	12 (7-35)	14 (7-34)	13 (7-35)	0.18
BMI (kg/m^2^)	31.1 ± 5.6	31.6 ± 5.3	31.4 ± 5.4	0.46
SBP (mm Hg)	137.5 (100-180)	140 (119-180)	140 (100-180)	0.14
DBP (mm Hg)	77.5 (50-90)	80 (60-95)	80 (50-95)	0.04
Hemoglobin (g/dl)	12.8 (8.8-16.2)	12.4 (7.3-15.9)	12.6 (7.3-16.2)	0.42
HbA1c (%)	7.1 (5.6-10.2)	7.3 (5.0-11.6)	7.2 (5.0-11.6)	0.23
Albumin (g/dl)	4.3 (3.4-4.9)	4.3 (3.2-5.0)	4.3 (3.2-5.0)	0.31
CRP (mg/dl**)**	0.20 (0-11.0)	0.20 (0-2.2)	0.20 (0-11.0)	0.68
Total cholesterol (mg/dl)	159 (103-234)	207 (112-345)	176 (103-345)	<0.0001
LDL cholesterol (mg/dl)	81.5 (41-137)	107.5 (54-245)	94.5 (41-245)	0.001
HDL cholesterol (mg/dl)	49 ± 13.8	46.9 ± 11.7	47.9 ± 12.8	0.50
Triglycerides (mg/dl)	120.5 (52-320)	174 (66-450)	140 (52-450)	0.001
EGFR (ml/min/1.73 m^2^)	71.3 (18.0-106.3)	49 (22.6-106.2)	59.6 (18.0-106.3)	0.013
UPCR (g/g)	0.13 (0.01-3.6)	0.17 (0.01-6.0)	0.15 (0.01-6.0)	0.2

*p* values of independent *t*-test and Mann–Whitney *U* test for differences of variables and *χ*^2^ test for differences in frequencies among groups. Ox-LDL: oxidized low-density lipoprotein; CV: cardiovascular; T2DM: type 2 diabetes mellitus; BMI: body mass index; SBP: systolic blood pressure; DBP: diastolic blood pressure; HbA1c: glycated hemoglobin A1c; CRP: C-reactive protein; HDL: high-density lipoprotein; eGFR: estimated glomerular filtration rate; UPCR: urinary protein to creatinine ratio.

**Table 2:** Cox proportional hazard analysis (forward stepwise regression) showing predictors for the combined end-point in DKD patients.

All-cause mortality or reduction of eGFR ≥ 30% or progression to ESKD				
	*B*	HR	95% CI	*p*
Model 1				
Ox − LDL > 66.22 U/L	1.23	3.42	1.55-7.56	0.002
Model 2				
Ox − LDL > 66.22 U/L	1.05	2.87	1.14-7.20	0.025
Baseline eGFR	-0.026	0.98	0.96-0.99	0.013

EGFR: estimated glomerular filtration rate; ESKD: end-stage kidney disease; HR: hazard ratio, CI: confidence interval; Ox-LDL: oxidized low-density lipoprotein; UPCR: urinary protein to creatinine ratio. Model 1: univariate model; model 2: multivariate model adjusted for baseline UPCR and eGFR, serum albumin, and triglycerides.

**Table 3:** Anthropometric, clinical, and biochemical data of patients with diabetic kidney disease, according to eGFR decline. Results for continuous variables are presented as mean (S.D.) or median (range).

	Slow decliners (*n*=39)	Fast decliners (*n* = 39)	*p*
History of CV disease (yes)	23/39	32/39	0.023
Duration of T2DM (years)	12 (7-28)	16 (7-34)	0.028
Triglycerides (mg/dl)	112 (52-320)	164 (53-450)	0.004
Ox-LDL (U/L)	59.5 (22.9-96.5)	75.3 (33.2-123.4)	0.005
UPCR (g/g)	0.12 (0.01-1.5)	0.14 (0.01-6.0)	0.013

*p* values of independent *t*-test and Mann–Whitney *U* test for differences of variables and *χ*^2^ test for differences in frequencies among groups. T2DM: type 2 diabetes mellitus; Ox-LDL: oxidized low-density lipoprotein; eGFR: estimated glomerular filtration rate; UPCR: urinary protein to creatinine ratio.

## Data Availability

The data used to support the findings of this study are included within the article.

## Supplementary Materials

Supplementary Materials Supplementary Table S1: multivariate Cox proportional hazard analysis (enter regression) showing predictors for the combined end-point in DKD patients (bootstrapping for 500 patients).
