# Supplementary material for: Oxidized LDL Is Associated with eGFR Decline in Proteinuric Diabetic Kidney Disease: A Cohort Study
Source: Oxid Med Cell Longev. 2021 Oct 19;2021:2968869. doi: 10.1155/2021/2968869 (PMC8548137; doi:10.1155/2021/2968869)
Supplement: Supplementary Materials — Supplementary Table S1: multivariate Cox proportional hazard analysis (enter regression) showing predictors for the combined end-point in DKD patients (bootstrapping for 500 patients). [file 2968869.f1.docx]

**Supplementary Table S1.** Multivariate Cox proportional hazard analysis (enter regression) showing predictors for the combined end-point in DKD patients (bootstrapping for 500 patients).

| **All-cause mortality or reduction of eGFR≥30% or progression to ESKD** | | | | |
| --- | --- | --- | --- | --- |
|  | *B* | *HR* | *95% CI* | *p* |
| Ox-LDL > 66.22 U/L | 0.99 | 2.70 | 1.02-7.16 | **0.046** |
| Baseline eGFR | -0.02 | 0.98 | 0.96-1.01 | 0.062 |
| Baseline UPCR | 0.04 | 1.04 | 0.73-1.47 | 0.85 |
| Serum Albumin | -0.39 | 0.68 | 0.25-1.83 | 0.44 |
| Serum Triglycerides | 0.0 | 1.00 | 0.99-1.01 | 0.87 |

*EGFR, Estimated Glomerular Filtration Rate; ESKD, End Stage Kidney Disease; HR, Hazard Ratio, CI, Confidence Interval; Ox-LDL, Oxidized Low-Density Lipoprotein; UPCR, Urinary Protein to Creatinine Ratio.*
